# Machine Learning‐Augmented Molecular Dynamics Simulations (MD) Reveal Insights Into the Disconnect Between Affinity and Activation of ZTP Riboswitch Ligands

**DOI:** 10.1002/anie.202505971

**Published:** 2025-06-22

**Authors:** Christopher R. Fullenkamp, Shams Mehdi, Christopher P. Jones, Logan Tenney, Patricio Pichling, Peri R. Prestwood, Adrian R. Ferré‐D'Amaré, Pratyush Tiwary, John S. Schneekloth

**Affiliations:** ^1^ Chemical Biology Laboratory National Cancer Institute Frederick MD 21702 USA; ^2^ Biophysics Program and Institute for Physical Science and Technology University of Maryland College Park MD 20742 USA; ^3^ Laboratory of Nucleic Acids National Heart, Lung, and Blood Institute, National Institutes of Health Bethesda MD USA; ^4^ Department of Chemistry and Biochemistry and Institute for Physical Science and Technology University of Maryland College Park MD 20742 USA; ^5^ University of Maryland Institute for Health Computing Bethesda Maryland 20852 USA

**Keywords:** Artificial intelligence‐augmented enhanced sampling, Medicinal chemistry, Molecular dynamics, RNA, Small molecule microarrays

## Abstract

The challenge of targeting RNA with small molecules necessitates a better understanding of RNA–ligand interaction mechanisms. However, the dynamic nature of nucleic acids, their ligand‐induced stabilization, and how conformational changes influence gene expression pose significant difficulties for experimental investigation. This work employs a combination of computational and experimental methods to address these challenges. By integrating structure‐informed design, crystallography, and machine learning‐augmented all‐atom molecular dynamics simulations (MD), we synthesized, biophysically and biochemically characterized, and studied the dissociation of a library of small molecule activators of the 5‐aminoimidazole–4–carboxamide ribonucleotide triphosphate (ZTP) riboswitch, a ligand‐binding RNA motif that regulates bacterial gene expression. We uncovered key interaction mechanisms, revealing valuable insights into the role of ligand binding kinetics on riboswitch activation. Further, we established that ligand on‐rates determine activation potency as opposed to binding affinity and elucidated RNA structural differences, which provide mechanistic insights into the interplay of RNA structure on riboswitch activation.

## Graphical abstract

This study combines computational and experimental approaches to study how natural and synthetic small molecules interact with the ZTP riboswitch, a bacterial RNA element that regulates gene expression. Using chemical synthesis, biochemical approaches, and artificial intelligence augmented MD simulations, we analyze both ligand binding and riboswitch activation. Findings show that ligand on‐rates, but not affinity, drive activation and offer insights into RNA structural dynamics.

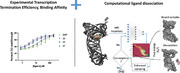

## Introduction

RNA is known to form complex secondary and tertiary structures with regulatory roles, such as influencing stability,^[^ ^1^, ^2^ ^]^ splicing,^[^ ^3^, ^4^, ^5^ ^]^ and gene expression.^[^ ^6^, ^7^, ^8^, ^9^ ^]^ Furthermore, folded 3D structures of RNA can form hydrophobic pockets^[^ ^10^ ^]^ that can be targeted with small molecules.^[^ ^11^, ^12^, ^13^ ^]^ As a result, RNA has reemerged as a therapeutic intervention point for diseases currently “undrugged” at the protein level.^[^ ^14^, ^15^, ^16^ ^]^ However, our knowledge about RNA–small molecule interactions and how small molecule binding influences RNA structure and function is limited compared to protein–ligand interactions. For example, a disconnect between binding affinity and activity in biochemical/biological assays for RNA ligands can be observed but is often difficult to rationalize, even when high‐resolution structures are available. Computational approaches that incorporate RNA dynamics could help address this challenge by successfully dovetailing experimental results with accurate, long‐timescale simulations.^[^ ^17^, ^18^, ^19^, ^20^, ^21^ ^]^

The regulatory functions of RNA depend on the interplay of metastable states within highly dynamic structural ensembles.^[^ ^22^ ^]^ Typically, biophysical methods developed for investigating protein–ligand interactions are adopted for studying RNA,^[^ ^23^, ^24^, ^25^ ^]^ but these methods are not always compatible with interrogating dynamic RNA–ligand interactions. However, advances in single‐molecule FRET assays,^[^ ^26^, ^27^, ^28^, ^29^, ^30^ ^]^ and computational methods have provided tools that enable the investigation of RNA structural dynamics^[^ ^31^ ^]^ and the discovery of small molecules targeting RNA dynamic ensembles.^[^ ^32^ ^]^ Molecular dynamics (MD) is a widely used computational method that has shown considerable success in studying biophysical problems of significance.^[^ ^33^ ^]^ Recent advances in computational hardware, including specialized supercomputers^[^ ^34^ ^]^ and GPUs^[^ ^35^ ^]^ have facilitated spatial parallelization and enabled the implementation of all‐atom MD for large systems. Such approaches can provide atomistic insights about transient phenomena that are elusive to experimental observations and aid in the development of RNA‐targeting small molecules. However, studying long time‐scale rare events such as ligand binding/unbinding events,^[^ ^36^ ^]^ and slow conformational changes in biomolecules^[^ ^37^ ^]^ can be challenging since simulations cannot be parallelized across time using improved hardware, and the time evolution of a system needs to be computed sequentially by solving equations of motion at each time step. Here, we applied machine learning‐augmented enhanced sampling to address this problem.^[^ ^38^ ^]^

Traditionally, the optimization of protein‐targeting small molecules has relied heavily on structure‐guided design using co‐crystal structures of protein‐ligand complexes.^[^ ^39^, ^40^ ^]^ However, compared to proteins, there are few co‐crystal structures of RNA–ligand complexes solved,^[^ ^41^ ^]^ and this has limited the use of structure‐guided design for optimizing RNA‐targeting molecules to far fewer instances.^[^ ^12^, ^32^, ^42^, ^43^, ^44^ ^]^ Bacterial riboswitches are a well‐understood class of structured mRNA motifs that control essential metabolic pathways for bacterial growth and virulence. Riboswitches control gene expression by sensing the intracellular concentration of a cognate ligand and, upon binding, undergo a conformational change that regulates gene expression.^[^ ^45^ ^]^ Riboswitches often have well‐defined 3D structures that contain hydrophobic pockets that can be targeted with drug‐like small molecules^[^ ^11^ ^]^ and have been implicated as potentially novel antimicrobial targets.^[^ ^46^ ^]^ Due to the well‐characterized functional effect that ligand binding has on gene expression and the availability of co‐crystal structures of riboswitch–ligand complexes, riboswitches are ideal model systems for investigating RNA–small molecule binding interactions and structure‐guided medicinal chemistry efforts.

In this work, we used structure‐informed design to synthesize a focused library of 27 small molecules that bind to and activate the *Fusobacterium ulcerans* ZTP riboswitch in vitro.^[^ ^47^ ^]^ However, upon further investigation we observed a poor correlation between in vitro riboswitch activation and ligand affinity among a library of eight novel ligands. To investigate this disconnect, we first co‐crystallized two ligands with the RNA and used machine learning‐augmented molecular dynamics simulations^[^ ^38^ ^]^ to investigate the diverse dissociation mechanisms of seven synthetic ligands and the cognate ligand 5‐aminoimidazole–4–carboxamide ribonucleotide monophosphate (ZMP). From these simulations for each ligand, we calculated the relative rate of ligand binding (*k*_on_), which is challenging to determine experimentally, and found that the values can distinguish between the synthetic, and cognate ligands. Furthermore, comparison of ligand dissociation trajectories identified key differences between the dissociation mechanisms of synthetic ligands compared to cognate ligand **ZMP**. These differences correlated with in vitro riboswitch activation and provide mechanistic insights into the role of flexibility, and specific riboswitch residues in the observed differences among ligands.

## Results and Discussion

### Structure‐Informed Design of *F. ulcerans* ZTP Riboswitch Binders

**Figure 1:**
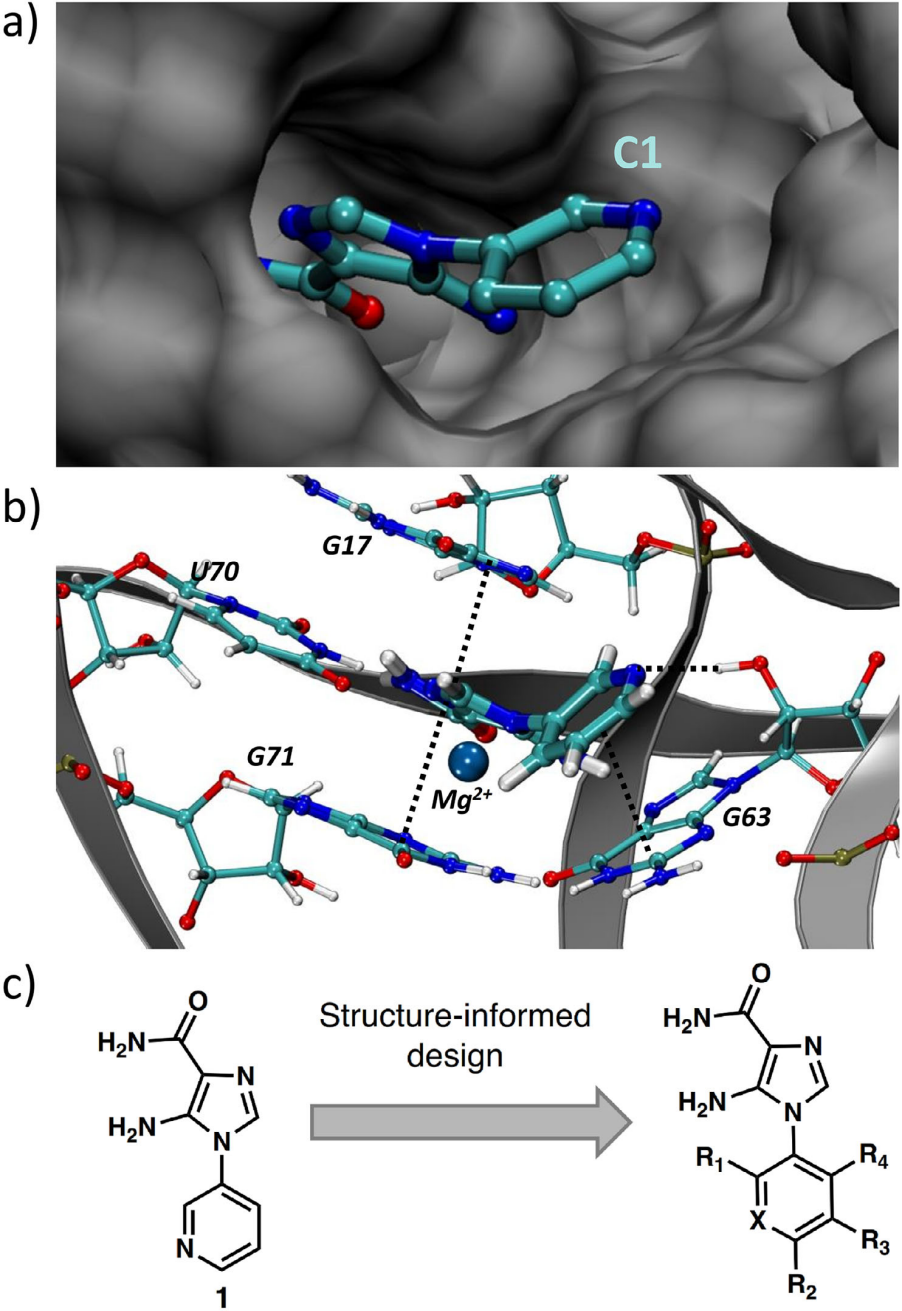
**Structural analysis of 1 bound to ZTP riboswitch and overview of the structure‐based design of synthetic binders of the** *F. ulcerans* **ZTP riboswitch**. a) The binding pose of **1** in complex with *F. ulcerans* ZTP riboswitch (PDB: 6WZS). Analysis indicates limited volume is available to accommodate additional modifications around the AICA core, and a potential cavity is present adjacent to the pyridine moiety of **1** (labeled C1), which could accommodate larger substituents. b) The binding mode of **1** highlights the key hydrogen bonding and π–π stacking interactions (black dotted lines) of **1** with bases U17, G17, G71, and G63. A proposed putative hydrogen bond interaction with the 2'‐OH of G63 and the pyridine moiety is highlighted with a purple dotted line. The blue sphere denotes a magnesium ion. c) Overview of the structural modification to **1** resulting in novel activators of *F. ulcerans* ZTP riboswitch highlighting the modifications made around the pyridine ring of **1**.

Previously, isosteric replacement of the phosphate and ribose sugar moiety of ZMP with a pyridine group resulted in the discovery of compound **1** (Figure 1c).^[^ ^48^ ^]^ Compound **1** possessed a weaker affinity (*K*_D_ ∼600 nM) but was a stronger riboswitch activator (*T*_50_ ∼5.8 µM) than cognate ligand ZMP (*K*_D_ ∼324 nM and *T*_50_ ∼37 µM) in biochemical assays.^[^ ^48^ ^]^ Here, $T_{50}$ indicates the concentration at which the riboswitch is half activated in single‐round transcription termination assays. Intrigued by the disconnect between ligand affinity and activation, we examined the reported co‐crystal structures of **ZMP** (PDB: 60D9) and **1** (PDB: 6WZS) bound to *F. ulcerans* ZTP riboswitch to rationally design new analogs. The binding pose of ZMP and **1** are very similar due to the conserved amino‐amidocarboxamide (AICA) core. Analysis of the co‐crystal structures indicated that due to the extent of burial of the imidazole core and the limited available volume, extensive modification of the AICA core would not be tolerated (Figure 1a). This observation is further supported by the inherent selectivity of the ZTP riboswitch for ZMP and ZTP over inosine, which is a downstream metabolic intermediate, and only a single carbon unit larger. Therefore, we directed our analysis to the solvent‐exposed area around the pyridine of **1**. ZMP and **1** each make unique interactions within the binding pocket and could result in the observed disconnect between affinity and activation. The ribose and phosphate moieties of ZMP make hydrogen bonds with the 2'‐OH of G63 and N4 of C69 (PDB: 60D9). In contrast, the pyridine moiety of **1** makes a π–π (black lines) stacking interaction with G63 and a putative hydrogen bond (purple line) with 2'‐OH of G63 (Figure 1b). The loss of the hydrogen bond interaction with N4 of C69 and gain of the π–π stacking interaction resulted in a ∼2‐fold loss in affinity for compound **1**, yet **1** had greater in vitro and in vivo activation of the *F. ulcerans* ZTP riboswitch.^[^ ^48^ ^]^ Additionally, adjacent to the pyridine group is a cavity (Figure 1a, **labeled C1**) that could accommodate larger substituents.

### Synthesis and Affinity Measurement of Designed Analogs

Based on the analysis of the co‐crystal structures of **ZMP** (PDB: 6OD9) and **1** (PDB: 6WZS) in complex with the *F. ulcerans* ZTP riboswitch, we designed and synthesized a library of 27 synthetic analogs that incorporated minor changes in 1) the AICA core or 2) the pyridine of **1** (Figure 1c)and Table 1, analogs **4–28**). The library of analogs was accessed by one of three synthetic routes. Compound **2** was afforded by cycloaddition of 3‐azidopyridine with 2‐cyanoacetamide in 30% yield.^[^ ^49^ ^]^ Compound **3** was accessed by nucleophilic aromatic substitution between 1H‐imidazole‐4‐carboxamide and 4‐chloropyridine in modest yields, and compounds **4–28** were synthesized by reaction of 2‐amino‐cyanoacetamide with triethyl orthoformate followed by the addition of substituted anilines, quinolines, or napthyridines to afford the desired analogs in modest to good yields^[^ ^48^, ^50^ ^]^ (Figures S1 and S2). With our library of analogs in hand, the equilibrium dissociation constant to the aptamer domain of *F. ulcerans* ZTP riboswitch was measured using isothermal titration calorimetry (ITC), following the previously described method.^[^ ^48^ ^]^

**Table 1:** Structure‐Informed Synthetic Analogs of m‐pyridinyl AICA and ITC *K*_*D*_.

Compound	Core	R	K_D_(µM)^a)^
ZMP	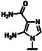	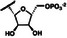	0.32 ± 0.13^c)^
1	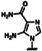	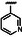	0.6 ± 0.06
2	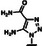	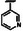	13.5 ± 7.85
3	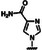	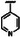	N.B.^b)^
4	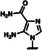	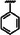	1.84 ± 0.41
5	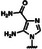	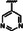	1.22 ± 0.45
6	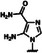	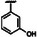	2.47 ± 0.37
7	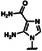	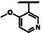	4.16 ± 0.68
8	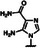	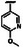	2.45 ± 0.44
9	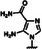	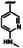	2.40 ± 0.36
10	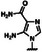	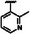	N.B.^b)^
11	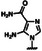	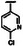	2.19 ± 1.4
12	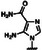	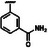	3.52 ± 0.60
13	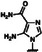	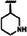	N.B.^b)^
14	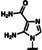	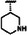	N.B.^b)^
15	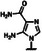	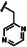	N.B.^b)^
16	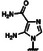	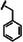	N.B.^b)^
17	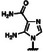	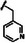	N.B.^b)^
18	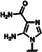	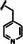	15.0 ± 7.1
19	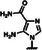	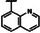	N.B.^b)^
20	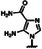	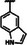	3.20 ± 1.3
21	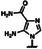	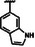	4.99 ± 0.64
22	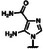	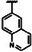	1.28 ± 0.26
23	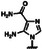	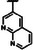	0.778 ± 0.28
24	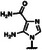	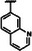	1.36 ± 0.17
25	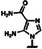	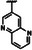	0.864 ± 0.21
26	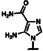	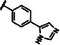	1.46 ± 0.21
27	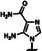	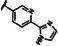	3.18 ± 0.44
28	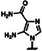	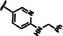	13.8 ± 1.5

^a)^ Values are mean ± standard deviation, with n = 3;

^b)^ N.B. indicates K_d_ > 30 µM or not measurable.;

^c)^ Value from Tran et al.

Because the AICA core was deeply buried in the binding pocket(Figure 1a), only minor modifications to the core were attempted. Single‐atom substitution (C to N) in the imidazole core, compound **2**, resulted in a ∼22‐fold loss in binding affinity (*K*_D_ = 13.7 ± 7.8 µM) compared to **1** (*K*_D_ = 0.60 ± 0.06 µM). In addition, the removal of the 5‐amino group, compound **3**, resulted in a complete loss of binding (*K*_d_ > 30 µM) (Table 1). These modifications highlight the importance of the AICA core for riboswitch–ligand recognition and binding. As AICA core modifications were not tolerated, we directed our efforts to replace the pyridine moiety of compound **1** with different aromatic and saturated ring systems. These efforts resulted in analogs with a range of affinities from ∼800 nM to >30 µM ((Table 1, **4–28**).

Replacement of the pyridine in compound **1** for a phenyl group, compound **4**, resulted in a ∼3‐fold loss of binding, highlighting the importance of the previously reported putative hydrogen bonding interaction between the nitrogen atom in the pyridine ring of **1** and the 2'‐OH of G63.^[^ ^48^ ^]^ The substitution of pyridine for pyrimidine, compound **5**, had a ∼2‐fold loss in affinity. In addition, replacement with piperidine, compounds **13** and **14**, resulted in a total loss of binding (*K*_D_ >30 µM), highlighting the importance of the π–π stacking interaction between the pyridine ring of **1** and G63. Furthermore, the substitution of the 2‐position of the pyridine ring with electron‐donating (methoxy (**8**) and N‐methylamine (**9**)) or electron‐withdrawing (Chloro (**11**)) resulted in a ∼4‐fold loss in affinity compared to **1** (Table 1).

Introduction of a methyl group ortho to the pyridinyl nitrogen atom of compound **1** or a methoxy group at position 4, compound **7**, to promote the ideal torsional angle for hydrogen bond formation with 2'‐OH of G63, abolished binding (Table 1, **7** and **10**, *K*_D_'s >30 µM). Further, replacement of the meta‐nitrogen of the pyridine with hydroxyl or an amide was tolerated but with a ∼5–6‐fold loss in binding affinity (**6** and **12**, Table 1). In addition, attempts to extend the ligand into cavity **C1** (Figure 1b), with benzylic aryl and quinoline moieties (compounds **15–19**), resulted in a ∼ 24‐fold reduction in affinity for **18** and complete loss of binding *K*_D_ >30 µM for **15–17** and **19**.

The extension of the pyridine moiety of **1** toward the opening of the binding cavity with quinolines (compounds **22** and **24**) and naphthyridines (compounds **23** and **25**) resulted in analogs with equivalent *K*_D_'s to **1** (*K*_D_ values of 0.778–1.38 µM vs. 0.610 µM, respectively). In contrast, replacement with an indole, **20** and **21**, resulted in a ∼7‐fold loss in affinity compared to **1**. Biaryl analogs (**26**, **27**, and **28**) also resulted in a loss of affinity. Overall, we rationally designed and synthesized a library of 27 new ligands and identified two ligands, **23** and **25**, with similar affinity to **1** (*K*_D_ ∼800 nM for **23** and **25** compared to *K*_D_ ∼610 nM for **1**). Our library highlights how small modifications to the ligand structure can result in drastic effects on binding affinities to *F. ulcerans* ZTP riboswitch. With our newly identified riboswitch binding analogs in hand, we next evaluated their ability to activate the *F. ulcerans* ZTP riboswitch in vitro.

### Synthetic Analogs Activate ZTP Riboswitch In Vitro

To evaluate the activation potential of our synthetic ligands, we conducted single‐round transcription termination assays with *F. ulcerans*m ZTP riboswitch using the previously reported methods.^[^ ^48^, ^51^ ^]^ Based on the regulatory mechanism of the *F. ulcerans* ZTP riboswitch, as an activator of transcription, we expect to see greater read‐through with increased binding of the RNA aptamer with ligand. The accumulation of the read‐through transcript can be quantified and fitted to determine *T*_50_, the concentration at which the riboswitch is half activated.

**Figure 2:**
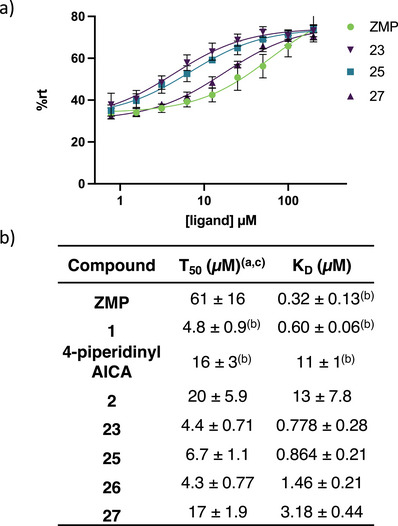
**Single‐round transcription termination efficiencies**. a) Single‐round transcription termination for **ZMP, 23, 25**, and **27** (*n*= 3, error bars denote standard deviations). b) Transcription termination efficiency (T_50_) and binding affinity (*K*_D_) for **ZMP**, **1**, **4‐piperidinyl AICA**, **2**, **23**, and **25–27**. Here, the superscripts correspond to: ^a^values reported as mean ± standard deviation, *n*=3; ^b^values obtained from Tran et al;^[^ ^48^ ^]^ and ^c^individual single‐round transcription termination titration curves for each analog are included in the Supporting Information.

ZMP was first retested and found to have a *T*_50_ in good agreement with the previously reported value (59 ± 15.8 µM vs. 37 ± 12 µM) (Figure 2).^[^ ^48^ ^]^ We then chose a subset of the newly designed synthetic ligands (**2, 23, 25, 26, 27** with a range of binding affinities (*K*_D_ ∼800 nM to 13 µM) to investigate their ability to activate transcriptional read‐through. Compounds **2, 23, 25, 26,** and **27** all had lower *T*_50_ values (i.e., better activation) than ZMP, even though ZMP is a tighter binder. These results are similar to the previously reported observation between **1**, **4‐piperidinyl AICA**, and **ZMP** (Figure 2),^[^ ^48^ ^]^ in which **1** and **4‐piperidinyl AICA** activate transcription to a greater extent than **ZMP**, even though both bind the riboswitch with a weaker affinity. A potential hypothesis explaining the observed disconnect between affinity and activation is riboswitch activation is driven by the rate of ligand binding (*k*_on_) and not overall affinity (*K*_D_). Using a single‐molecule FRET‐based assay to study riboswitch folding, it was previously demonstrated that ZMP's activation of the ZTP riboswitch is driven by *k*_on_;^[^ ^29^ ^]^ however, it has not been shown that this holds true across a panel of synthetic ligands.

### Investigation of Binding Kinetics by Surface Plasmon Resonance

To investigate the role ligand binding kinetics of our synthetic analogs have on riboswitch activation, experimental determination of *k*_on_ for our synthetic ligands was attempted using surface plasmon resonance (SPR) experiments with the aptamer domain of the *F. ulcerans* ZTP riboswitch. Using both the traditional streptavidin reference channel subtraction method^[^ ^52^ ^]^ and the recently reported non‐binding mutant RNA reference channel subtraction method,^[^ ^25^ ^]^ we observed ligand binding of compound **26** with the aptamer domain of *F. ulcerans* ZTP riboswitch and obtained *K*_D_ values of ∼ 900 nM, which is in good agreement with our ITC value (*K*_D_ ∼ 1.46 ± 0.21 µM, Figure S2). However, the plotted response curve did not reach saturation and exceeded the theoretical max response for a 1:1 binding event by more than 2‐fold, presumably due to non‐specific binding or aggregation (Figure S2). In addition, *k*_on_ and *K*_off_ for **26** could not be extracted from the SPR response curves due to the observed steep slope for association and dissociation (Figure S2). Since kinetic information could not be obtained experimentally, we set out to investigate ligands via co‐crystallography and use those models to facilitate the study of ligand binding kinetics by MD methods.

### Co‐Crystal Structures of Compounds **1** and **23** Bound to the *S. odontolytica* ZTP Riboswitch

**Figure 3:**
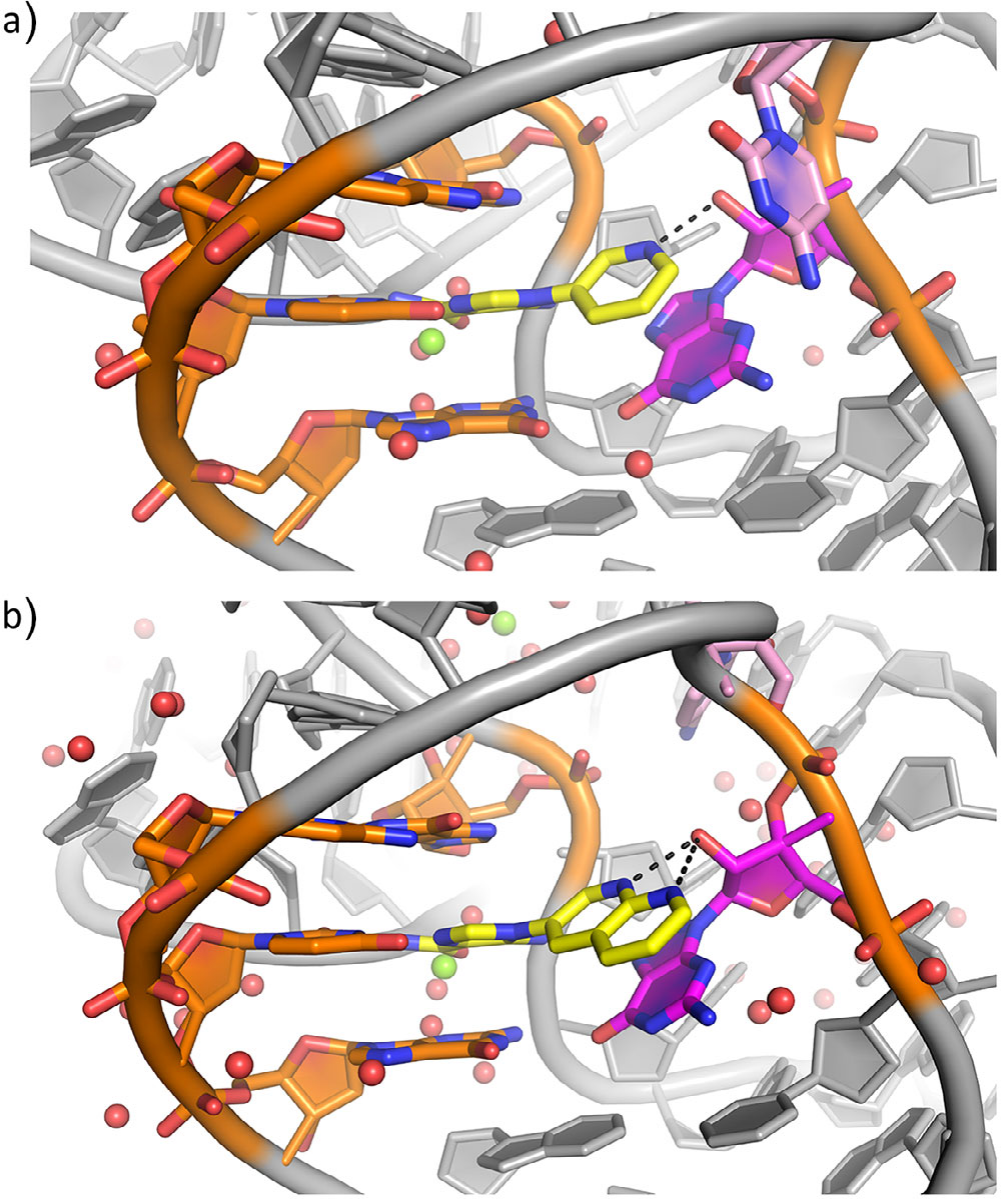
**Co‐crystal Structures of ZTP riboswitch in complex with synthetic analogs highlight conserved binding mode**. a) Co‐crystal structure of compound **1** yellow with *S.ondontolytica* ZTP riboswitch. The black dotted line indicates hydrogen bonding interaction with G51(magenta). b) Co‐crystal Structure of Compound **23** (yellow) with *S. ondontolytica* ZTP riboswitch. The black dotted line indicates potential hydrogen bonding interactions between G51 (magenta) and the N1 and N8 of the napthyridine of **23**. In this model, C52 (pink) undergoes a conformational change, moving away from the napthyridine of **23**. Green spheres denote magnesium ions, and red spheres denote waters.

Our previously reported structure of compound **1** was determined at 3.2 Å resolution bound to the *F. ulcerans* ZTP riboswitch;^[^ ^48^ ^]^ thus, we sought to validate those findings in the *S. odontolytica* ZTP riboswitch, which has been reported to crystallize at higher resolution. We co‐crystallized **1** with the *S. ondontolytica* ZTP riboswitch aptamer, solved the structure via molecular replacement, and refined the structure to 2.4 Å resolution (Table S1, Methods). In the model, the pyridine moiety is poised to hydrogen bond to the 2'‐OH of G51 (3.0 Å, Figure 3a), consistent with our previous findings. Nearby C52 is about 4.0 Å away from the pyridinyl group and hydrogen bonds with non‐bridging phosphate oxygen (NBPO) O2 of C50 (3.0 Å). In the presence of ZMP, a hydrated magnesium ion makes inner sphere contacts with C52 (Figure 3a).

In contrast to **1**, for which co‐crystals were readily obtained, larger synthetic ligands proved more challenging. We co‐crystallized **23** to the *S. ondontolytica* RNA in different conditions, habits, and space groups (Methods). The structure determined at 2.2 Å resolution was partially solved and refined due to the presence of weakly resolved copies in the asymmetric unit (Table S1). As in **1**, **23** is poised to hydrogen bond with the 2'‐OH of G51 (2.9 Å) via the N1 of **23**, and the AICA moiety is bound as for **1** (Figure 3b). An additional hydrogen bond is also possible between N8 of **23** and the 2'‐OH of G51 (3.2 Å). However, likely due to steric clashing with the larger ligand, C52 no longer hydrogen bonds to NBPO O2 and instead pairs with G34, which is unpaired in the presence of ZMP and **1**. In addition, in the presence of **23**, loop residues A16‐19 undergo a conformational change that is involved in making crystal contacts. The conformation of the residue after G51 varies among ZTP riboswitch sequences. Consequently, its conformation participating in an H‐bond with C50 (i.e., as in Figure 3 and ref. [53]) has only been observed in *S. odontolytica*.

### Insights into RNA–Ligand Interaction Mechanisms Using All‐Atom Molecular Dynamics (MD)

As the experimental apo‐structure remains unresolved and conducting an accurate MD simulation of the association event is prohibitively expensive due to the substantial associated barrier,^[^ ^54^ ^]^ we simulated and investigated dissociation events instead. We employed the experimentally solved crystal structure (PDB: 6WZS) of ZTP riboswitch bound to **1** ^[^ ^48^ ^]^ as the initial structure for all simulations. This structure has 15 missing residues (1–5, 48–54, 57–59), which we modeled using ChimeraX.^[^ ^55^ ^]^ Afterwards, we obtained bound structures for all the other ligands by swapping out **1** and using template‐based molecular docking within Molsoft ICM‐Pro.

The recent DES‐Amber forcefield^[^ ^56^ ^]^ was used to parametrize the RNA, and the small molecules were parametrized using GAFF2.^[^ ^57^ ^]^ The authors of DES‐Amber developed improved ion parameters specifically for $Mg^{2+}$ to help balance affinity between water and negatively charged phosphates and carboxylates groups on proteins and nucleic acids^[^ ^56^ ^]^ that were employed in this work. The developers aimed to keep the ionic radius similar to experimental values while maintaining high accuracy, which was achieved by rescaling the charge by a factor of 0.9. Furthermore, DES‐Amber developers^[^ ^56^ ^]^ performed extensive benchmark simulations and ab initio calculations to demonstrate its applicability for RNAs and structure‐stabilizing $Mg^{2+}$ ions.

The six synthetic derivatives (**compounds 2, 23, 25, 26, 27**) studied in this work are charge neutral, and thus the results from GAFF2 were kept unchanged. Since the phosphate group of ZMP loses two protons at physiological conditions, we modeled ZMP accordingly, and after generating the GAFF2 forcefield, we rescaled the partial charge of the phosphate group by a factor of 0.9 to be compatible with the DES‐AMBER forcefield. We took a similar approach when parametrizing the only positively charged derivative (**4‐piperidinyl AICA**). We note that in previous studies,^[^ ^58^ ^]^ it has been reported that the electronic properties of $Mg^{2+}$ ion can depend on the type of interacting ligands. However, all of the ligands studied in this work have the exact same polar group with which $Mg^{2+}$ ion interacts, and thus have the same binding motif. The bound structures were then solvated using 30234 TIP4P‐D^[^ ^59^ ^]^ water molecules and neutralized using $Na^{+}$, and $Cl^{-}$ ions by maintaining 0.15 M concentration to mimic the physiological conditions of the experiments. We performed all the simulations at 303.15 K temperature and 1 atm pressure using the Nose–Hoover thermostat^[^ ^60^ ^]^ and Parinello–Rahman barostat,^[^ ^61^ ^]^ respectively. To investigate the suitability of the adopted force fields for our systems, we performed 180 ns of unbiased simulation after energy minimization and equilibration for each of the ligands and observed stable behavior between $Mg^{2+}$–water, $Mg^{2+}$–RNA, and $Mg^{2+}$–ligand interactions (Figures S3–S6).

**Figure 4:**
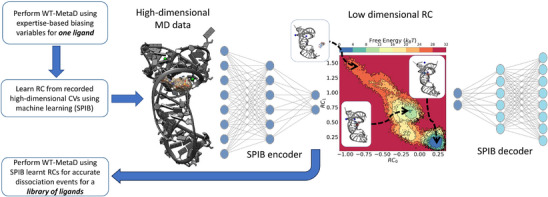
**Overview of machine learning (SPIB) augmented MD framework**. Variational autoencoder implementation of SPIB learns a low‐dimensional reaction coordinate (RC) from high‐dimensional MD data for one ligand, which was then used as the biasing variable of WT‐MetaD for a library of ligands. The top left and bottom right portions of the plot represent unbound and bound states, respectively.

We summarize our protocol for simulating the dissociation process in Figure 4. We used the first 50 ns of the unbiased simulation as the initial step and recorded the standard deviation of all the collective variables (CVs) (see Supporting Information) for one of the ligand–RNA complexes (**compound 26**). Afterward, well‐tempered metadynamics^[^ ^62^, ^63^, ^64^, ^65^, ^66^, ^67^ ^]^ (WT‐MetaD) as an enhanced sampling algorithm is implemented only for this ligand to observe dissociation by biasing $d_{MG^{2+}-l_{1}}$, and $d_{RNA_{17}-l3}$ distances, which point along the cavity exit. We took 75% of the standard deviation of the unbiased run as WT‐MetaD σ, with height =1.5 kJ ${\mathrm{mol}}^{-1}$, bias factor = 40, pace = 2.0 ps. Without using such a high σ we were unable to observe dissociation in this round, indicating the need for more accurate biasing variables.

We addressed this by learning an improved, meaningful description of RNA–ligand interactions by implementing state predictive information bottleneck^[^ ^64^ ^]^ (SPIB), a machine‐learning^[^ ^68^ ^]^ (ML) framework for identifying low‐dimensional reaction coordinates (RCs) as the linear combination of high‐dimensional CVs from the above simulation for a single ligand, which was then used to implement subsequent rounds of WT‐MetaD for accelerating MD simulations for a ligand library consisting of eight ligands chosen for computational studies (**ZMP, 1, 4‐piperidinyl AICA, 2, 23, 25, 26,** and **27**). We chose **compound 26** since it's a large synthetic ligand with an end‐to‐end distance comparable to **ZMP**, allowing the SPIB model to learn RNA–ligand interactions happening in the outer parts of the cavity as well as on the inside. By transferring the model, we were able to avoid training models for each individual ligand, which makes this protocol scalable. We observed between 12−16 independent dissociation events for each ligand using ML‐assisted WT‐MetaD with 25% of the standard deviation of the SPIB reaction coordinates (RCs) in the bound state used as σ, height =1.5 kJ ${\mathrm{mol}}^{-1}$, bias factor = 40, pace = 2.0 ps. Additional discussions on SPIB theory and model training details are provided in the Supporting Information.

**Figure 5:**
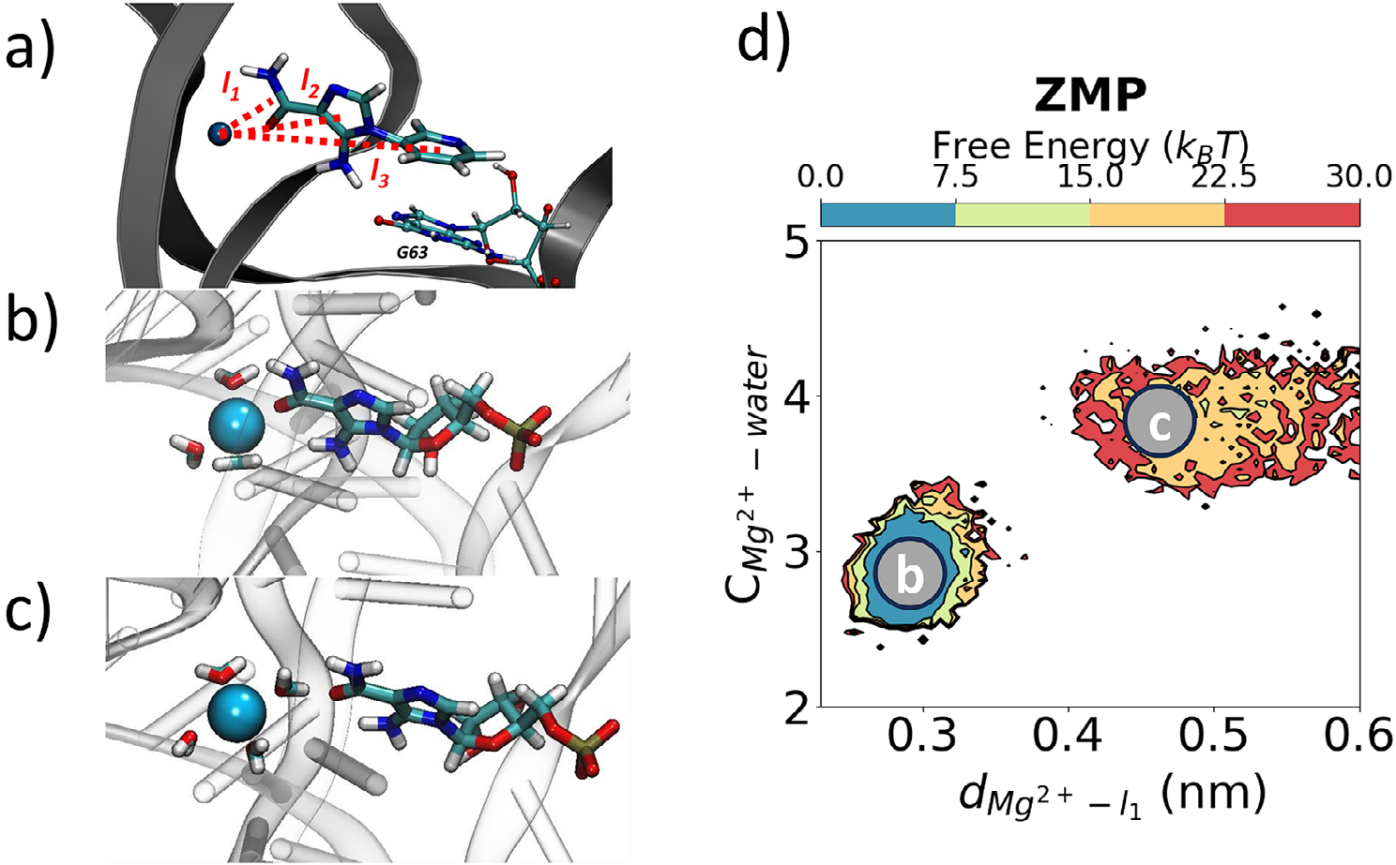
**Change in structural** $Mg^{2+}$ **coordination during ligand dissociation**. a) Definition of the $l_{1}$, $l_{2}$, $l_{3}$ distances between $Mg^{2+}$ ion and center of masses of the polar, inner core, and outer moieties of the ligands, respectively. b) Free energy projections of water‐$Mg^{2+}$ distance as **ZMP** exits. c,d) $Mg^{2+}$ ion hydration as the ligand exits. The structures correspond to the labels in panel (b).

**Figure 6:**
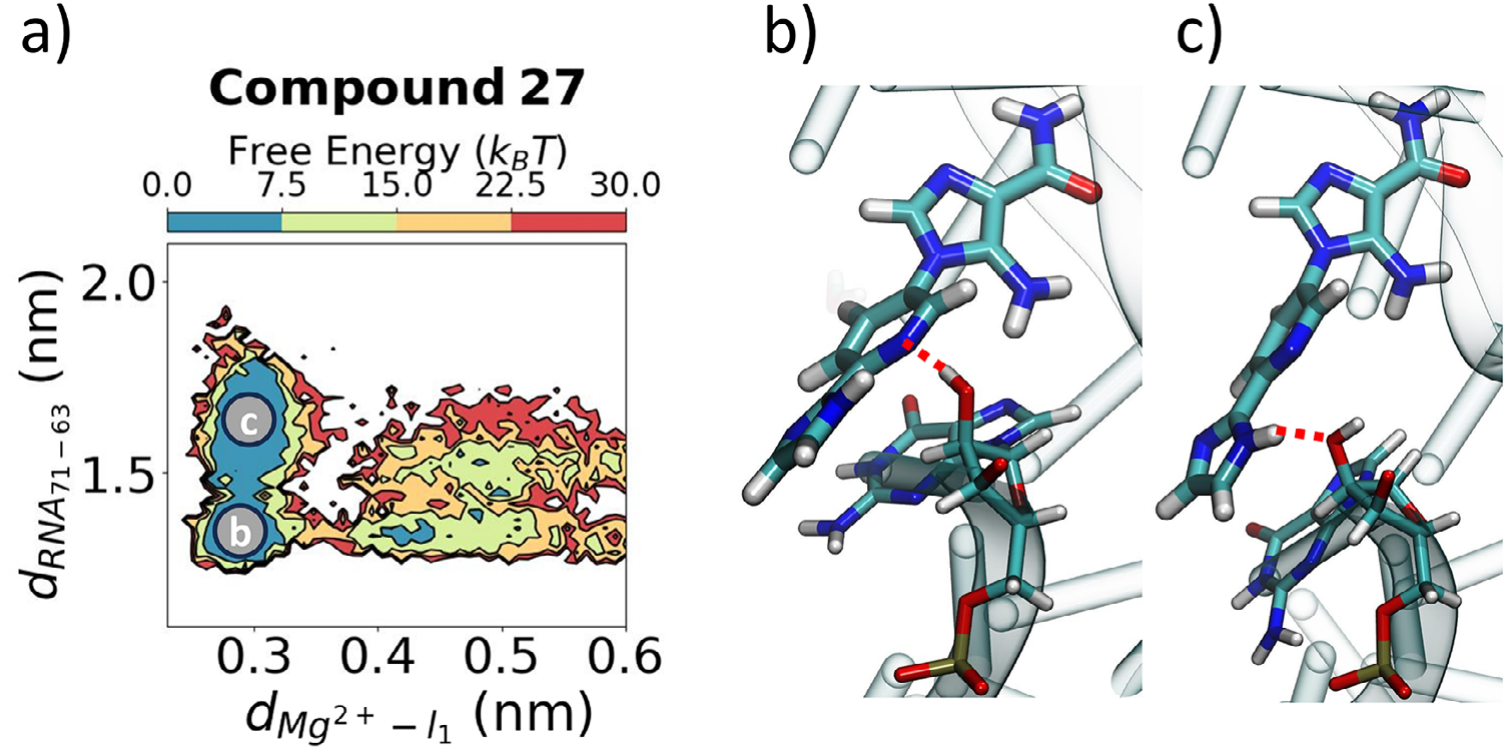
**Synthetic derivatives exhibit different behavior in the bound conformation**. a) Free energy projected along the distance between the backbone of G71 and nucleobase of G63 (*y*‐axis) as **compound 27** exits the binding site, as measured by the $Mg^{2+}$–ligand distance. In the bound conformation, there are two metastable states, highlighted in (b,c) showing alternating π−π stacking and hydrogen bonding interactions with G63 between the outer ligand moieties. The red dotted lines in panels (b,c) indicate hydrogen bonds.

**Figure 7:**
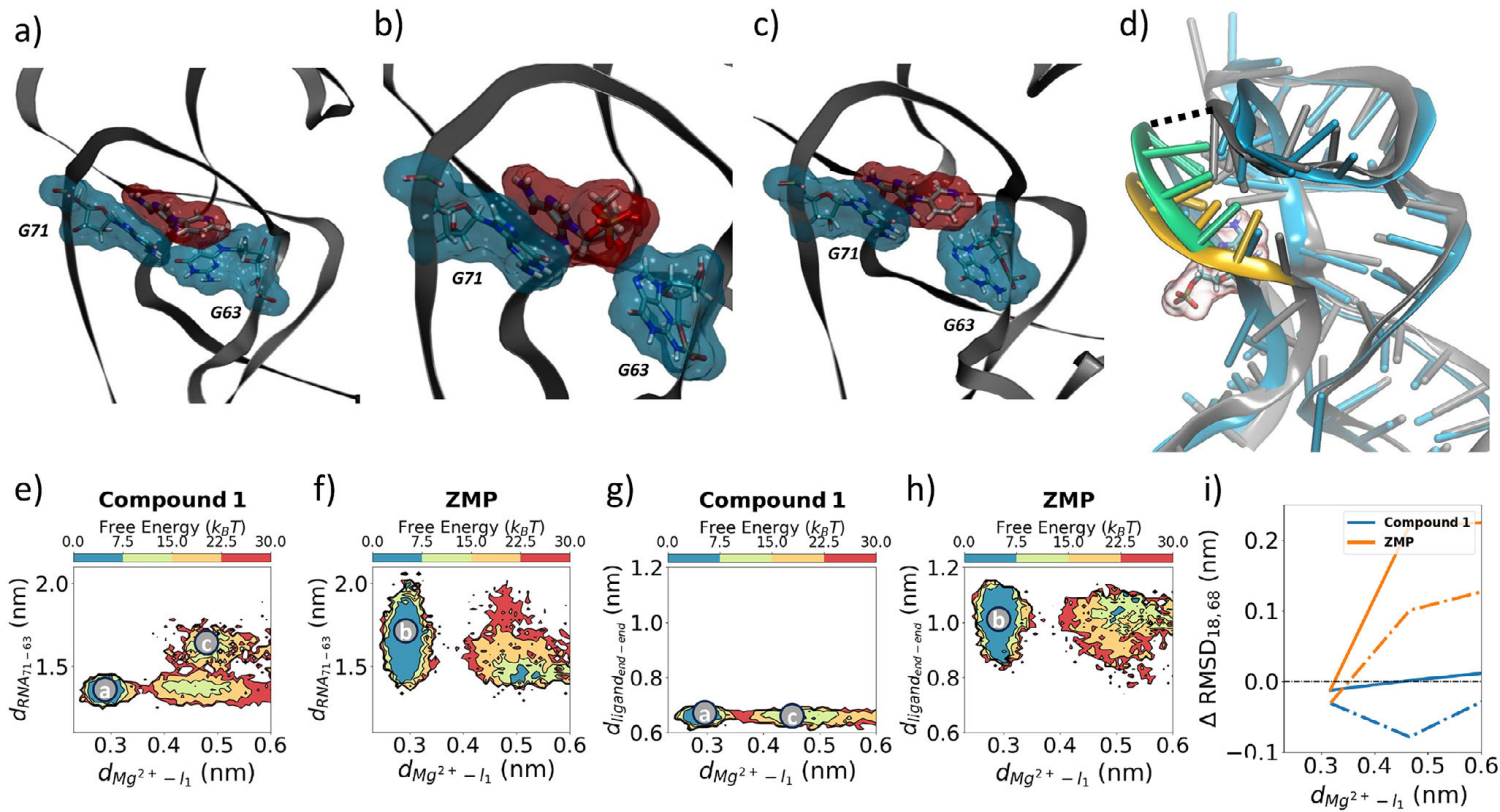
**MD simulations of RNA–ligand interactions**. a) **Compound 1** bound to ZTP riboswitch with pyridine moiety exhibiting π−π stacking interactions with G63. In this conformation, RNA residues G63 and G71 (highlighted using cyan clouds) are in close proximity. b) **ZMP** bound to ZTP riboswitch with RNA residues G63 and G71 (highlighted in cyan) separated, c) dissociation of **1** involves opening of the G63 nucleobase to allow ligand exit, d) increased flexibility of the P4 domain (green, and yellow) of ZTP riboswitch during ligand dissociation. Two specific residues in the P4 domain (A18 and U68) are highlighted with a dotted line. Free energies are projected on the distance between the backbone of G71 and nucleobase of G63 as the ligands exit for e) **1** and f) **ZMP**, respectively. In the bound conformation (panels a,c), the spread in this distance is smaller for **1** compared to **ZMP**. As the ligand exits (panel c), this distance increases for **1**. End‐to‐end distance for g) **1**, and h) **ZMP**, respectively. The rotatable bonds present in **ZMP** allow higher flexibility. i) Δ RMSD for A18 (solid line), and U68 (dotted line) show that **ZMP** induces significant flexibility to P4 domain compared to **1** during exit.

Analysis of the final dissociation trajectories unraveled key mechanistic details about the individual RNA–ligand interactions as depicted in Figures 5, 6, 7. In the bound conformation for the ligands, the RNA structural $Mg^{2+}$ ion was observed to coordinate with three water molecules (Figure 5b,c), the amide of the ligands, and with the phosphate groups of U16 and C35, respectively. Eventually, a fourth water molecule coordinates with the $Mg^{2+}$ ion, leading to a loss of interaction with the ligands, allowing for dissociation (Figure 5d).

After decoordination between the $Mg^{2+}$ and the oxygen atom of the amide moiety, the dissociation mechanism differs between **ZMP** and synthetic ligands (**1, 4‐piperidinyl AICA, 2, 23, 25, 26, 27**). Figure 7a shows the pyridine ring of **compound 1** forms a π−π stacking interaction with G63, allowing G63 and G71 to remain in close proximity via a noncanonical interaction (Figure 7e). However, for **ZMP**, the sugar moiety sits between G63 and G71 (Figure 7b,f). When the synthetic ligands exit the cavity (Figure 7c), the distance between the backbone of G71 and nucleobase of G63 increases (Figure 7c,e). Here, we noted that this increase in distance between G63 and G71 for the synthetic ligands occurs after the hydration of the $Mg^{2+}$ ion, as discussed previously. However, for **ZMP**, as the ligand exits, the average distance between G63 and G71 decreases (Figure 7f). In this case, after the hydration of $Mg^{2+}$, the volume available to **ZMP** in the cavity decreases, and **ZMP** undergoes an end‐to‐end contraction before exiting the cavity (Figure 7g,h).

The ligands exit the cavity through a pathway close to the P4 domain (Figure 7d,i). We observed **ZMP** inducing higher flexibility in this domain compared to the synthetic derivatives. This is quantified by Δ RMSD (root‐mean‐square deviation), which is defined as the change in RNA–ligand distance as the ligand exits when compared to a long (180 ns) unbiased simulation of a ligand‐removed structure. Larger derivatives (**23, 25–27**) also induced higher flexibility to a few of the residues in the P4 domain, but to a lesser extent than observed for **ZMP**. A detailed summary of Δ RMSD calculations for all residues and for each ligand is provided in the Supporting Information (Figures S9 and S10).

In addition to the differences between **ZMP** and the synthetic derivatives computationally studied in this work, synthetic derivatives also displayed distinct behavior. **Compound 27** (**23 and 25** to a lesser extent) exists as two metastable states in the bound state (Figure 6). We designed **Compound 27** with both a hydrogen bond donor and acceptor with the intention of introducing a bivalent hydrogen bond interaction. However, we observed two metastable states where the π−π stacking interaction alternates between the inner pyridine and outer imidazole groups due to the formation of distinct hydrogen bonds in the bound state highlighted by dotted lines in (Figure 6b,c).

### Computational Results Distinguish between Ligand Classes

**Figure 8:**
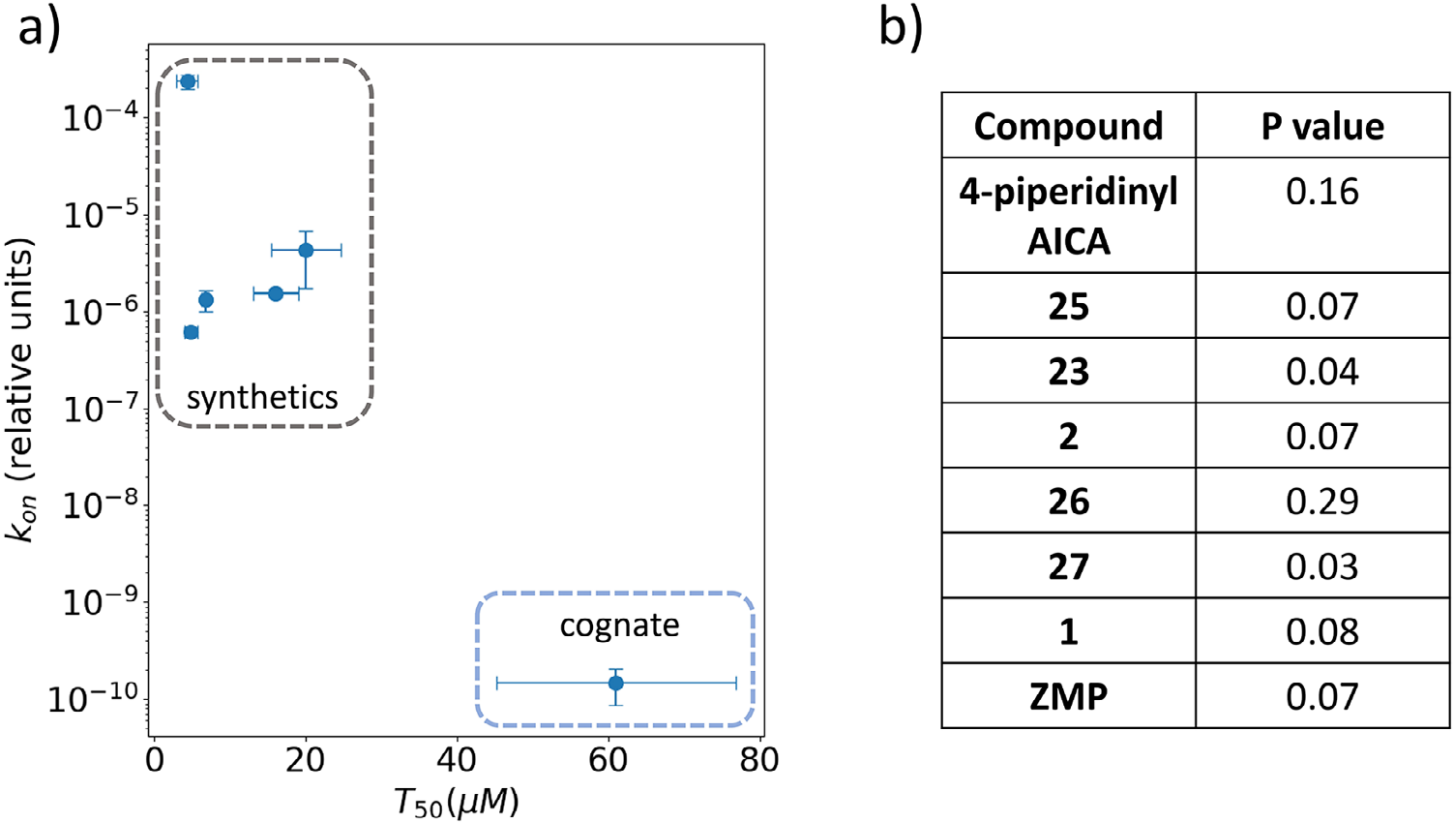
a) MD‐derived relative $k_{on}$ for the transferred model trained on **compound 26**. $T_{50}$ error bars represent experimental standard deviation, while $k_{on}$ errorbars were computed by first calculating the theoretical standard deviation of $t_{\mathrm{rel}}$ from MD simulations and then using error propagation formula to incorporate experimental $K_{D}$ error, b) *P* value associated with KS test.

In addition to investigating RNA–ligand interaction mechanisms, we used the theory of infrequent metadynamics (iMetaD)^[^ ^62^ ^]^ and the same WT‐MetaD trajectories obtained above to compute relative residence times ($t_{\mathrm{rel}}$). Here, we note that our goal is not to compute exact residence times for the individual ligands, which would be highly challenging due to the use of a transferred model but to distinguish between cognate and synthetic ligand classes. The transition time (t) to go from bound (A) to unbound (B) state was estimated by computing the associated acceleration factor,^[^ ^62^ ^]^ $\alpha \left(t\right)={\left\langle e^{\beta (V(s(R,t)))}\right\rangle }_{s\in A}$. Here, we defined the bound state by setting a cut‐off of 6.33 Å on the $Mg^{2+}-l_{2}$ distance, corresponding to the dominant free energy barrier associated with hydration of the $Mg^{2+}$ ion and disruption of the G71‐inner core stacking of the ligands (Figure S8). Using experimentally determined $K_{D}$, and computational rate of dissociation ($k_{{\mathrm{off}}_{\mathrm{rel}}}=1/t_{\mathrm{rel}}$) we computed the relative rate of association of the ligands ($k_{{\mathrm{on}}_{\mathrm{rel}}}$) using the relation, $K_{D}=\frac{k_{{\mathrm{off}}_{\mathrm{rel}}}}{k_{{\mathrm{on}}_{\mathrm{rel}}}}$. From Figure  8a, we see differences in the computed values between the synthetics and cognate ligand classes. According to theory,^[^ ^62^ ^]^ the statistic of the computed values coming from independent dissociation trajectories should follow a Poisson distribution. In panel (b) of this figure, the *P* values associated with the Kolmogorov–Smirnov (KS) test^[^ ^69^, ^70^ ^]^ show that six out of eight ligands (including **ZMP**) satisfy this requirement (P>0.05) and give us confidence about the results. Thus, in panel (a), we plotted $k_{{\mathrm{on}}_{\mathrm{rel}}}$ for the six ligands that passed the KS test. Since we performed WT‐MetaD instead of iMetaD, obtaining a reliable estimate of error bars for $t_{rel}$ can be challenging due to limited statistics and the presence of outliers, and in this case, we used the *P* values of the KS tests to check the reliability of the results. Still, we used a parametric approach for computing error in $t_{\mathrm{rel}}$ by taking the square root of the mean of the fitted Poisson curve representing theoretical standard deviation and then used error propagation to obtain errorbars for $k_{{\mathrm{on}}_{\mathrm{rel}}}$. Finally, to test the robustness of this entire protocol, we trained a separate SPIB model for **compound 27** using an expertise‐based dissociation trajectory and transferred the model to all the ligands to simulate between 10−20 independent dissociations for each. The calculated $k_{{\mathrm{on}}_{\mathrm{rel}}}$ for this transferred model agrees closely with that of **compound 26**. Detailed results of this model are provided in the Supporting Information (Figure S15).

## Conclusion

In this work, we demonstrated how structure‐informed design can be used to identify new ligands that bind to and activate the *F. ulcerans* ZTP riboswitch in vitro. We identified analogs **23**, **25**, and **26**, which have equivalent affinities (*K*_D_ ∼800 nM, Table 1) and in vitro riboswitch activation (*T*_50_ ∼5 µM, Figure 2) as the previously reported analog **1** (*K*_D_ ∼610 nM and *T*_50_ of 4.8 µM).^[^ ^48^ ^]^ We did not identify analogs with improved affinity or activation than **1** in part due to the limited volume available for modification around the AICA core and the solvent accessibility of the binding cavity around the pyridine moiety of **1** (Figure 1a). Although the C1 pocket offers a direction for such improvement, this region also varies among the solved structures.^[^ ^53^, ^71^, ^72^ ^]^ Access to crystal structures of RNA bound to ligands is frequently difficult to acquire, and in this work crystallography was only successful with an aptamer from the *S. ondontolytica* ZTP aptamer, which has a similar ligand binding site. Although an imperfect comparison, the structural work demonstrates that the new compounds bind via a consistent mode and coordinate to the conserved magnesium ion. An additional complexity specific to ZTP riboswitches is the complex and kinetically controlled folding landscape of the aptamer. Multiple assays suggest that in solution the aptamer is often unfolded, meaning that a crystal structure is insufficient to completely describe the ensemble of conformations adopted by the RNA and the development of novel strategies may be required to apply structure‐based design. The difficulty in identifying novel analogs with improved affinity has also been encountered with other riboswitch systems.^[^ ^13^ ^]^ However, from our synthetic endeavor, we gained insights into the type of modifications that promote riboswitch‐ligand interaction. We observed that aromatic and heteroaromatic rings can act as suitable isosteric replacements for the ribose and phosphate moiety of **ZMP**, which is consistent with previously reported studies for the ZTP riboswitch^[^ ^48^ ^]^ and FMN riboswitch.^[^ ^11^, ^73^ ^]^ In addition, from our molecular dynamics simulations, we observed two metastable states for the bound conformation of compound **27** (**23** and **25** to a lesser extent). We intended with the design of compound **27** to shift the hydrogen bonding interaction from a hydrogen bond acceptor from pyridine in **1** to a hydrogen bond donor from the NH of the imidazole in **27**, and from our simulations, we were able to observe this binding mode shift without obtaining a co‐crystal structure.

Furthermore, a disconnect between ligand affinity and riboswitch activation was observed for our synthetic analogs (**2**, **23**, and **25–27**) and **ZMP** (Figure 2.) Our synthetic ligands (**2**, **23**, and **25–27**) all displayed greater in vitro activation than cognate ligand **ZMP** even though their *K*_D_s are ∼2–20‐fold weaker. The disconnect is hypothesized to result from differences in the *k*_on_ of ligand, the rate of transcription, and the rate of RNA unfolding.^[^ ^74^, ^75^ ^]^ Unfortunately, after multiple different attempts our efforts to experimentally acquire on rates using surface plasmon resonance were unsuccessful. The *F. ulcerans* ZTP riboswitch undergoes co‐transcriptional folding and senses the intracellular concentration of cognate ligand **ZMP** over an ∼5–10 nucleotide window; it is expected that riboswitch activation would be influenced more by *k*_on_ than *K*_D_ since activation occurs too quickly for binding to reach equilibrium.^[^ ^29^, ^76^, ^77^ ^]^ Although it is difficult to unambiguously demonstrate this mechanism, our work is consistent with a fast ligand on rates driving activity rather than aptamer folding kinetics. Our molecular dynamics (MD) simulations allowed for the calculation of relative ligand binding kinetics. Additionally, we gained insights into the differences in RNA structural dynamics during ligand dissociation.

Unlike previous studies which used MD simulations to analyze only the RNA by itself^[^ ^78^ ^]^ or RNA‐cognate ligand,^[^ ^29^ ^]^ we performed a comparative study of our synthetic ligands and **ZMP**, and found key differences in their dissociation trajectories (Figure 7). The two primary differences are the extension of the P4 domain upon ZMP exiting the cavity and the behavior of G63‐G71 distance. Residues in the P4 domain are part of the interdomain pseudoknot that forms the binding pocket but also overlap with the terminator hairpin sequence. The ZTP riboswitch undergoes a ligand‐gated competitive strand displacement to form the terminator hairpin during transcription, and the binding of ZMP stabilizes the pseudoknot, disfavoring terminator hairpin formation.^[^ ^76^ ^]^ We hypothesize that the differences in the G63‐G71 distances and the observed extension of the P4 domain during ZMP dissociation may promote or aid internal strand displacement and termination hairpin formation. As G63 and G71 compose part of the ligand binding pocket and are essential for ZMP binding,^[^ ^71^ ^]^ the G63‐G71 distance likely reports on both ligand binding and P4 stability. A caveat here is that the native RNA chain grows during transcription, so the RNA that initially binds to the ligand may be shorter than the RNA that promotes dissociation, for example, by destabilizing P4. The nuanced nature of structure–reactivity relationship trends in this system highlights the complexity of RNA–ligand interactions. For example, observed alterations in RNA–ligand hydrogen bonding and stacking interactions are not obviously reflected in changes in affinity but do manifest in measurable activity changes. This could be explained by multiple effects, such as altered hydration of the conserved magnesium or ligand flexibility, as described above.

From a computational perspective, we trained our ML model on the dissociation trajectory of one ligand (**compound 26**) and implemented the learned reaction coordinate (RC) to accelerate the dissociation of all the other ligands. Here, the successful dissociations beyond **compound 26** demonstrate that our approach was able to construct a transferable model. To check the robustness of the protocol, we implemented the same transferred model approach for **compound 27** and observed similar results (Figure S15). As far as we know, this is the first application of ML‐augmented MD simulation for enhancing the sampling of RNA‐ligand interactions where the model can be generalized to study a variety of ligands using state‐of‐the‐art RNA forcefields^[^ ^56^ ^]^ with atomistic detail. Such a framework would be broadly useful in both rationalizing complex and nuanced structure‐activity relationship trends often seen with RNA‐binding ligands as well as designing novel, improved ligands for other therapeutically relevant RNA targets, efforts that are currently ongoing in our labs.

In conclusion, we demonstrated the utility of using computational and experimental tools to study and understand ZTP riboswitch activation by small molecules and how these tools can be used to investigate the mechanism of riboswitch activation. The insights learned from our MD simulations about RNA–ligand interactions and the observed ligand‐induced structural differences could be used in future research for the design of ligands that leverage both binding kinetics, G63‐G71 distance, and P4 destabilization for the design of inhibitors of *F. ulcerans* ZTP riboswitch, potentially serving as antimicrobial agents. Taken together, this methodology could be applied to prospective studies aimed at harnessing ligand association/dissociation and conformational dynamics in the design of more potent, bioactive ligands for disease‐relevant RNAs.

## Conflict of Interests

The authors declare no conflict of interest.

## Supporting information

Supporting Information

## Data Availability

The data that support the findings of this study are available from the corresponding author upon reasonable request.
